# Incidence of Cochlear Implantation Complications in Saudi Arabia: A Comprehensive Systematic Review of the Literature

**DOI:** 10.7759/cureus.60488

**Published:** 2024-05-17

**Authors:** Bassam AlRajhi, Muhnnad A AlGhamdi, Noura Alenazi, Haila Alabssi, Sham T Alshammeri, Qusay Aloweiny, Hassan Bogari, Haya Al-Subaie

**Affiliations:** 1 College of Medicine, King Saud Bin Abdulaziz University for Health Sciences, Jeddah, SAU; 2 College of Medicine, King Saud Bin Abdulaziz University for Health Sciences, Riyadh, SAU; 3 College of Medicine, Imam Abdulrahman Bin Faisal University, Dammam, SAU; 4 College of Medicine, University of Hail College of Medicine, Hail, SAU; 5 Otolaryngology Head and Neck Surgery, National Guard Hospital Jeddah, Jeddah, SAU

**Keywords:** systematic review, saudi arabia, otology, complications, cochlear implants

## Abstract

This review aimed to determine the incidence of complications associated with cochlear implants (CI) in Saudi Arabia. We systematically searched PubMed, AIRE, OaIster, MEDLINE, Directory of Open Access Journals, Scopus, and Ovid from inception to January 2024. The review protocol was registered in the International Prospective Register of Systematic Reviews (PROSPERO) (ID: CRD42023486687). Studies that reported CI complications in Saudi Arabia were included. This systematic review was conducted in accordance with PRISMA guidelines. A total of 17 articles with 2216 patients were included. The most common intraoperative complication was cerebrospinal fluid leakage (23 patients, 0.99%), followed by surgical difficulties (six patients, 0.26%), and dural accidental exposure and bleeding (three patients, 0.13%); the most common postoperative complications were vestibular symptoms (20 patients, 0.86%), followed by infection (17 patients, 0.73%), and device malfunction or migration (12 patients, 0.52%). The total complication rate ranged from 4-13%. Most of the included studies had a low risk. CI in Saudi Arabia has a complication rate similar to that reported in international studies. This review emphasizes the need for continued surveillance of CI outcomes to optimize procedural techniques and improve the safety and efficacy of CI in Saudi Arabia.

## Introduction and background

Cochlear implants (CI) are surgically implanted devices designed for people with advanced sensorineural hearing loss (SNHL), a condition that refers to an improper functioning of the tiny hair cells inside the inner ear or a damage to the auditory nerve. This impairment is common, affecting approximately 466 million people globally, making it the most common issue related to our senses [1]. CI changes regular sounds into electrical signals, directly exciting the auditory nerve within the spiral ganglia. This process helps individuals deal with severe hearing loss because it allows them to perceive sounds and speech. CIs are considered a great alternative to traditional hearing aids [1]. Recently, many children struggling with hearing loss have been undergoing CIs, with an average of 78% of hearing-impaired children undergoing this procedure [2]. However, this scenario changes significantly for adults, where only 6.6% of eligible candidates opt for CIs [2]. The factors contributing to this difference between adults and children remain unclear and require further investigation. One factor that could contribute to this difference is the routine screening of hearing loss among newborns, which has become a mandatory practice as part of a clinical program to detect hearing loss in the early stages in many countries.

Although CIs bring positive transformations, they also have a spectrum of potential complications. These include intra and post-operative complications such as infections, extrusion, facial nerve stimulation, meningitis, and cerebrospinal fluid leaks. The likelihood of encountering these complications is linked to factors such as the inherent complexity of the surgical procedure, surgical skills of the operating surgeon, and their experience with the specifics of cochlear implantation [3].

In light of the current advancement in medical technologies related to cochlear implants in Saudi Arabia, a comprehensive systematic review study is needed to evaluate these operations and advancements and to determine the incidence of cochlear implant complications. Thus this systematic review aimed to determine the incidence of complications after CIs in Saudi Arabia which can provide valuable insights for clinical practices and policymakers to assist otolaryngologists and otologists in providing the best preoperative and postoperative care for patients undergoing a cochlear implantation surgery.

## Review

Materials and methods

Overview

The study protocol was registered in the International Prospective Register of Systematic Reviews (PROSPERO) (ID: CRD42023486687). This review was conducted in accordance with the Preferred Reporting Items for Systematic Reviews and Meta-Analyses (PRISMA) 2020 updated guidelines [4].

Eligibility Criteria

All individuals of all age groups and both sexes who had undergone cochlear implantation in Saudi Arabia were included, regardless of the occurrence of complications. The main reason for including studies with and without complications was to ensure proper reporting of the rate and incidence of CI complications. Studies that did not report any outcomes were excluded. Restrictions to non-English studies were applied, and further assessment of studies was carried out to exclude duplicate studies.

Information Sources

We performed a systematic literature search of PubMed, AIRE, OaIster, Medline, Directory of Open Access Journals, Scopus, and Ovid, from inception to January 2024. No timeframe restrictions were imposed. The records were exported from the databases using Excel sheets. Our search terms included (cochlear OR cochlear implants) AND (complications) AND (Saudi Arabia). We used Medical Subject Headings (MeSH), EMTREES, and keywords to develop a comprehensive search strategy. We also complemented our search strategy with a manual search by reviewing the references of all included studies.

Selection Process

Two investigators (NFA and SA) independently screened the titles and abstracts of the selected databases. Full-text assessment of eligibility using predefined criteria was performed independently by two investigators. Any disagreement between the two investigators was resolved by the lead authors (BA and MAG) or consensus.

Data Extraction

Data extraction was independently performed by two investigators (HOB and HA). Data were extracted from eligible articles using a data collection sheet designed by one of the authors (HA). Variables extracted from the articles included study characteristics, such as study design, study duration, sample size, total CIs, study setting, mean age, gender, study limitations, and study conclusions. Participants’ characteristics were extracted and included the cause of hearing loss, site of CI, type of CI device, average follow-up duration, and intraoperative and postoperative complications.

Risk of Bias Assessment

The risk of bias assessment was performed by two investigators (NFA and QA). The Methodological Index for Non-randomized Studies (MINORS) was used. Any disagreement between the two investigators was resolved by the lead authors (BA and MAG) or consensus.

Results

**Figure 1:**
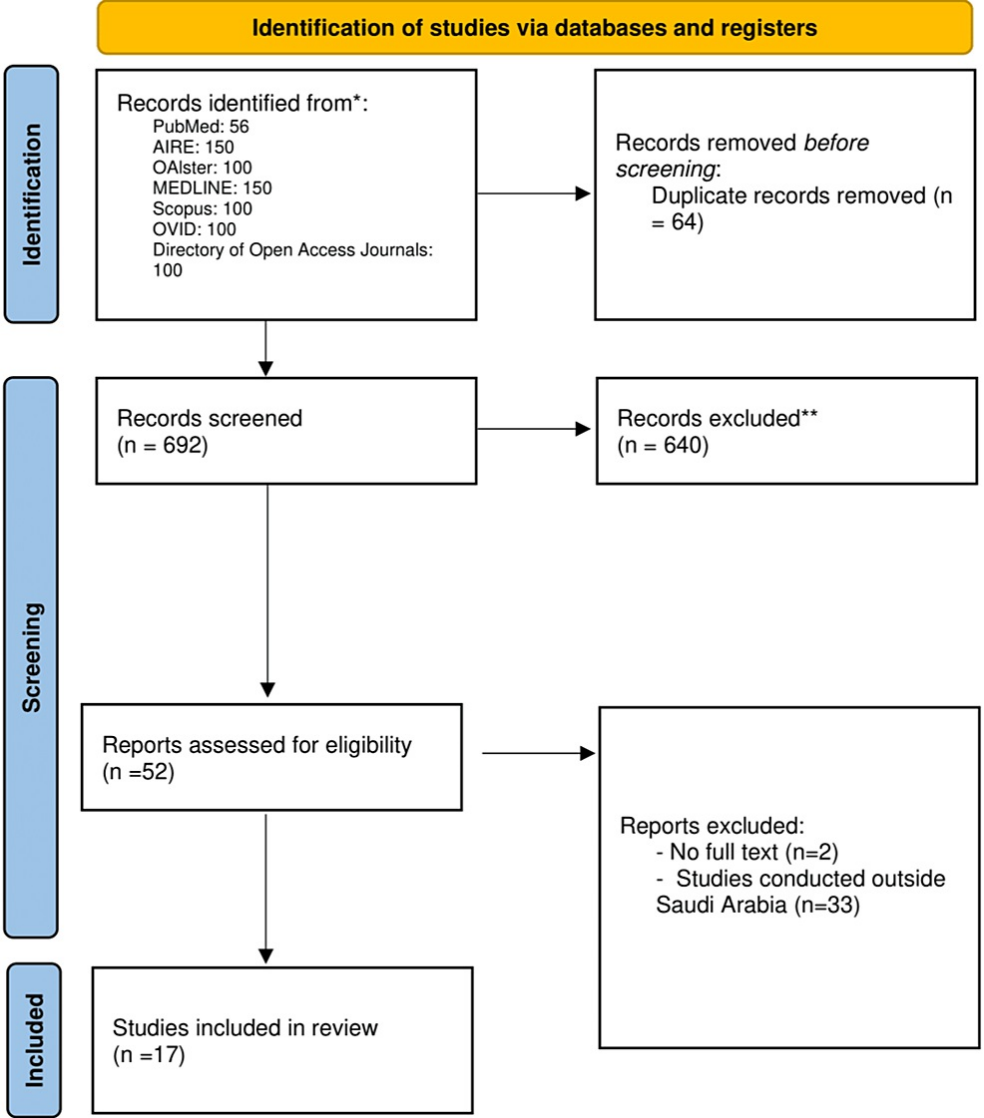
The PRISMA flowchart showing the screening process of articles based on the study inclusion and exclusion criteria PRISMA: Preferred Reporting Items for Systematic Reviews and Meta-Analysis

The literature search yielded a total of 756 articles. The articles were screened for duplicates, and 62 articles were excluded at this stage. Screening by title and abstract was performed and 640 articles were excluded. The remaining 52 articles underwent a full-text assessment. Of these, 35 articles were excluded because they were conducted outside Saudi Arabia, and two were excluded because of the lack of a full-text article. Finally, 17 articles published between 2007 and 2024, with 2216 patients, were included in this systematic review (Figure 1).

Characteristics of the Included Studies

Of the included studies, 10 were retrospective [5-14], one was a cross-sectional study [15], three were case series [16-18], and three were case reports [19-21]. All studies were conducted in Saudi Arabia. A total of 366 patients were males and 302 were females. However, gender was not reported in five studies. Pediatric patients were included in six studies, with a mean age ranging from 10 months to 9 years, and the mean age for adults ranged from 17 to 66 years. The study duration was reported in eight studies (42.1%), with an average of 8.75 years. The total number of CIs reported from all included studies was 2314. The causes of hearing loss have been reported in nine studies.

The most common cause of hearing loss among patients who underwent CI was idiopathic hearing loss (175 patients, 7.89%). This was followed by congenital and cochleovestibular malformation, accounting for 36 patients each, followed by non-specific febrile illness in 24 patients, trauma in 18 patients, and meningitis in 16 patients. Other causes of hearing loss included presbycusis in seven patients, cholesteatoma in five, chronic suppurative otitis media in four, and drug-induced hearing loss in three. Brucellosis infection was reported to be the cause of hearing loss in one patient. In one case, hearing loss was reported to be secondary to bilateral temporal bone fractures and part of the Vogt-Koyanagi-Harada syndrome. The site of CI was reported in some studies, with 174 patients having left-sided implants, 110 patients having right-sided implants, and 57 patients having bilateral implants. The average follow-up duration was reported in only two studies, five and three years. Further details are presented in Table 1 and Table 2.

**Table 1:** Characteristics of the included studies and patients’ demographics NR: Not reported, NA: Not applicable

Study	Study design	Study duration	Total population	Total cochlear implants	Settings/location	Mean age (in years)		Gender	
						Children	Adults	Males	Females
Alsughayer, 2020 [20]	Case report	2 years	1	1	King Abdullah Ear Specialist Center	2	NR	1	0
Halawani, 2020 [8]	Retrospective	NR	177	177	Tertiary health care center	3	31	98	79
Alzhrani, 2018 [10]	Retrospective	4 years	200	200	King Abdullah Ear Specialist Center	NR	NR	NR	NR
Al-Muhaimeed, 2009 [11]	Retrospective	12 years	117	117	King Abdulaziz University Hospital	NR	NR	70	47
Alhelali, 2019 [19]	Case report	NR	1	2	NR	NA	30	0	1
Halawani, 2019 [9]	Retrospective	11 years	1027	1027	Tertiary health care center	NA	NR	NR	NR
Yousef, 2022 [12]	Retrospective	NR	7	14	King Abdullah Ear Specialist Center	9	17-66	6	1
Al-khatib, 2023 [13]	Retrospective	4 years	167	167	King Abdulaziz University Hospital	4.49 ± 3.07	NR	79	88
Arab, 2012 [18]	Case series	NR	3	NR	University Hospital	6	0	1	2
Hagr, 2007 [21]	Case report	NR	1	1	NR	NR	48	0	1
Alshaikh, 2019 [15]	Cross-sectional	NR	13	246	King Fahad General Hospital, Jeddah	6	0	12	1
Garrada, 2021 [14]	Retrospective	20 years	148	162	King Abdulaziz University Hospital (KAUH)	NR	NR	73	75
Fatani, 2023 [6]	Retrospective	13 years	27	NR	NM	NR	37 ± 15.5	16	2
Almofada, 2023 [17]	Case series	4 years	131	NR	King Faisal Specialist and Research Hospital	NR	NR	NR	NR
Hajr, 2019 [7]	Retrospective	NR	15	24	Tertiary referral center	0.8	0	10	5
Aljazeeri, 2021 [5]	Retrospective	NR	144	176	King Abdullah Ear Specialist Center	NR	35 ± 15	NR	NR
Aldhafeeri, 2021 [16]	Case series	NR	37	NR	NR	2.5	43.6	NR	NR

**Table 2:** Characteristics of studies’ participants, intervention, follow-up duration, limitations and conclusion of the included studies NR: Not reported, SNHL: Sensorineural hearing loss, CVM: Cochleovestibular malformation, TBF: Temporal bone fracture.

Study	(N) Causes of hearing loss	Site of implant (N)			Type of cochlear implant device	Average follow-up duration	Study limitations	Study conclusion
		Right	Left	Both				
Alsughayer, 2020 [20]	SNHL (1)	NR	NR	1	NR	NR	NR	Tip fold-over is an uncommon complication of CI. Revision surgery should be reserved for patients with persistent symptoms or significantly affected speech performance, where deactivation did not suffice.
Halawani, 2020 [8]	CVM (36)	92	85	0	NR	NR	NR	The form 24 array can help surgeons to stop intraoperative, CSF, gusher and prevent postoperative CSF leakage and meningitis in Cochlear implant recipients with a CVM.
Alzhrani, 2018 [10]	NR	NR	NR	NR	NR	NR	NR	Pediatric patients with otitis media can safely and effectively undergo cochlear implants in one stage.
Al-Muhaimeed, 2009 [11]	Unknown (95) Congenital (17). Chronic suppurative otitis media (4). Meningitis (1)	NR	NR	NR	Multichannel Cochlear Implants devices	NR	NR	Outcomes are comparable to the previous reports, with a note on the possibility of an abnormally related cochlea in case of difficulty during cochleostomy
Alhelali, 2019 [19]	Vogt-Koyanagi-Harada Syndrome (1)	0	0	1	NR	NR	Limited by the nature of a single case report	Cochlear implantation in patients with Vogt-Koyanagi-Harada Syndrome can be challenging due to intra-cochlear fibrosis, but it can provide good hearing outcomes.
Halawani, 2019 [9]	NR	NR	NR	NR	NR	NR	Single center	CI is considered safe, emphasizing the importance of preoperative protocol, surgical technique, and postoperative care
Yousef, 2022 [12]	Bilateral temporal bone fractures (1)	NR	NR	1	NR	NR	NR	CI is a safe surgical procedure with potential benefits for individuals with bilateral TBF
Al-khatib, 2023 [13]	NR	NR	NR	NR	NR	NR	Retrospective design, variations in surgeon experience	Well-Drilling technique is faster; no significant difference in safety
Arab, 2012 [18]	NR	NR	NR	2	NR	NR	NR	CI extrusion can be managed with local scalp flaps, and ideal results can be achieved by ensuring effective soft tissue coverage using technique suitable to the clinical presentation.
Hagr, 2007 [21]	NR	NR	NR	2	Clarion 90 K	NR	NR	This is the first report in the literature of delayed onset as well as asymptomatic pneumocephalus after cochlear implantation.
Alshaikh, 2019 [15]	Meningitis (13)	13	NR	NR	Cochlear™ Nucleus® (5) and MED-EL (8)	NR	Small sample size	Cochlear implantations are safe for patients with meningitis-induced deafness, with no surgical complications reported.
Garrada, 2021 [14]	NR	NR	NR	14	Cochlear 59, MED-EL 92, Advanced Bionic 11	5 years	NR	Cochlear implantation is deemed safe and dependable, with a minimal rate of complications when conducted by skilled surgeons.
Fatani, 2023 [6]	Meningitis (2, 7.4%). Trauma (3, 11.1%). Non-specific high-grade fever (3, 11.1%). Brucellosis infection (1, 3.7%). Unknown (18, 66.7%)	NR	NR	27	NR	NR	Retrospective study design potentially affecting of accuracy data collection, and a relatively small sample size.	Simultaneous bilateral cochlear implants can be offered to adult patients with no intraoperative complications or lengthy recovery periods.
Almofada, 2023 [17]	NR	NR	NR	NR	NR	NR	NR	The transmeatal approach carried an increased rate of complications on long-term follow-up including formation of cholesteatoma, extrusion of electrode or peri-electrode reaction formation to tympanic membrane and external auditory canal.
Hajr, 2019 [7]	NR	5	1	9	NR	3 years	Presence of missing data, specifically the amount of blood loss, due to the retrospective nature of the research and the relatively limited sample size.	This research constitutes the most extensive national group of pediatric patients receiving cochlear implants during infancy. The findings indicate that the surgical procedure was safe, and the speech outcomes were positive. The introduction of a neonatal screening program in Saudi Arabia is expected to boost the number of infants undergoing cochlear implantation in the coming years, potentially leading to further investigations in this area.
Aljazeeri, 2021 [5]	Drug-induced (3) Presbycusis (7) Cholesteatoma (5) Traumatic (15) Febrile Illness (21) Congenital progressive (19) Unknown (63) Other (5)	NR	80	NR	NR	NR	Retrospective design	Cochlear implants done for post-lingual adults yielded significant improvements in auditory performance with low complication rates.
Aldhafeeri, 2021 [16]	NR	NR	NR	NR	Med-El = 693 (20 of them experienced device failure) Cochlear= 200 (8 of them experienced device failure) Advance Bionic = 29	NR	The study’s limitation needs to compare more different CI manufacture to evaluate more for devices reliability.	CI surgery is increasingly common, necessitating long-term follow-up protocols for CI recipients due to potential complications that can arise. Addressing declines in patient performance requires a multidisciplinary approach, as recommended in the present study. Efforts from companies are crucial to minimize instances of device failure.

Intraoperative Compilations

**Figure 2:**
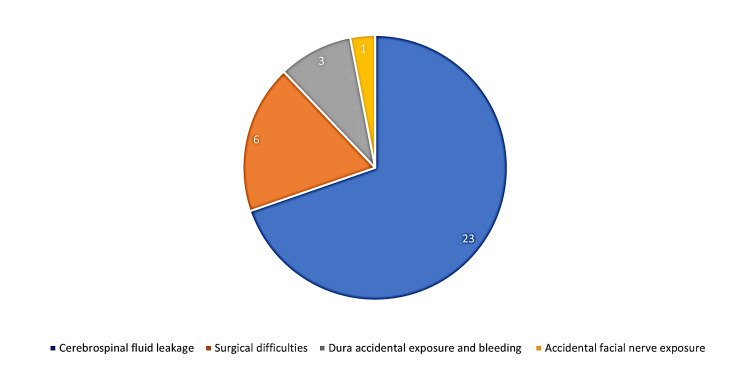
Percentage of intraoperative complications of cochlear implants as reported by the included studies

CI complications can be classified into major and minor complications. Major complications were defined as events that led to additional surgical, medical, or invasive outpatient interventions. Minor complications are events that are self-limiting, can improve on their own, or require minimal conservative treatment. Minor intraoperative complications of CI include cerebrospinal fluid (CSF) leakage, which was the most reported intraoperative complication observed in a total of 23 patients (0.99%). Surgical difficulties were reported in six patients (0.26%), either due to resistance to inserting an electrode due to causes such as intracochlear fibrosis, or due to other factors such as weakness of the posterior canal, injury to the auditory canal, and pain that required conversion to general anesthesia. Aljazeeri et al. reported that dura accidental exposure and bleeding occurred in a total of three patients (0.13%) [5]. Aljazeeri et al. also reported accidental facial nerve exposure in one case [5]. No major intraoperative complications associated with CIs have been reported. The distribution of intraoperative complications is shown in Figure 2.

Postoperative Complications

**Figure 3:**
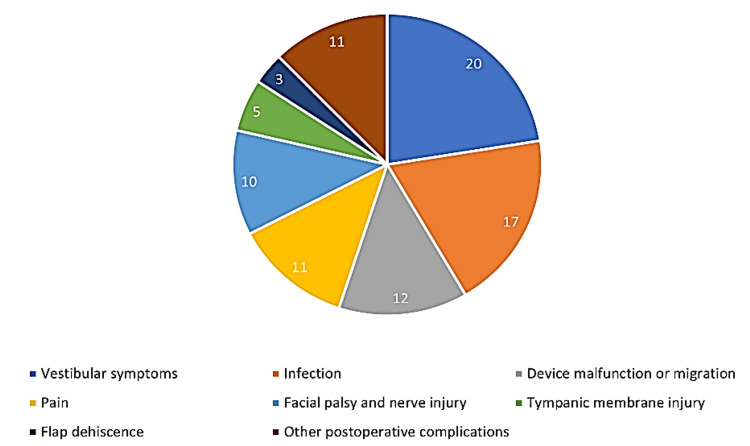
Percentage of postoperative complications of cochlear implants as reported by the included studies

Postoperative complications had the same classification system as intraoperative complications. There were several minor complications of CI, the most reported postoperative complication was vestibular symptoms in 20 patients (0.86%), followed by infection in 17 patients (0.73%), and device malfunction or migration was reported in 12 patients (0.52 %). Pain was reported in 11 patients (0.47%). Major postoperative complications included facial palsy and nerve injury, which were reported in 10 patients (0.43 %). Other major postoperative complications, such as intra-parenchymal pneumocephalus, change in taste sensation, cholesteatoma, keratin deposition, and other causes that required revision surgery were found in 11 (0.48%) patients. Tympanic membrane injury was observed in five patients (0.22%), and flap dehiscence was reported in three patients (0.12%). The distribution of postoperative complications is shown in Figure 3.

A total of 119 patients (5.14%) had non-specific complications that were not mentioned by the authors of the included studies. The total rate of complications was 4-13%, with a lower rate of complications (4%) reported by Aldhafeeri et al. [16], and the highest rate of complications (33.3%) reported by Fatani et al. [6]. We excluded the case reports reported by Hajr et al. [7] and AlHelali et al. [19], in which one patient had a 100% rate of complications.

Risk of Bias Assessment

**Figure 4:**
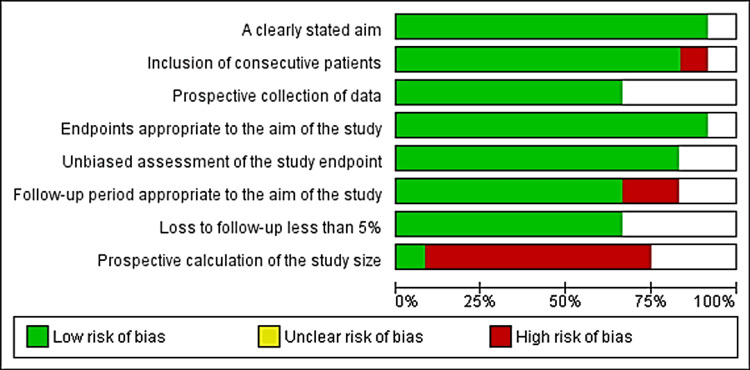
MINORS instrument assessment for non-randomized non-comparative studies (N=12) 0 = not reported, 1 = reported but inadequate, 2 = reported adequately A total score of 16-22 indicates a low risk of bias, 8-15 indicates a moderate risk of bias, and 0-7 indicates a high risk of bias. MINORS: Methodological Index for Non-Randomized Studies

**Figure 5:**
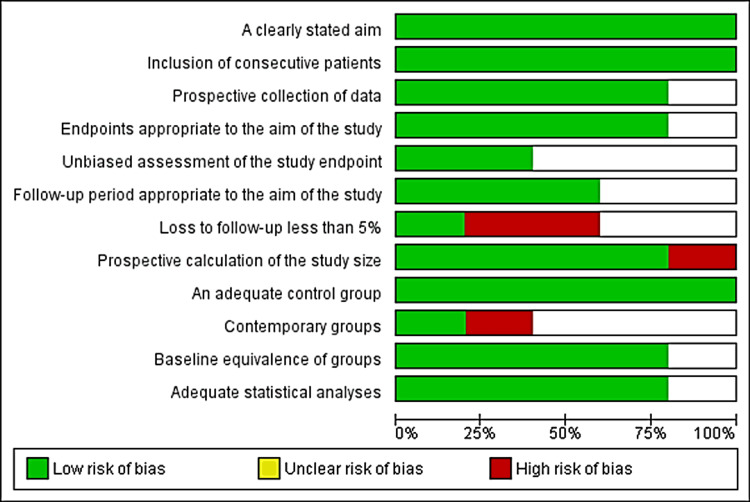
MINORS instrument assessment for non-randomized comparative studies (N=6) 0 = not reported, 1 = reported but inadequate, 2 = reported adequately A total score of 16-22 indicates a low risk of bias, 8-15 indicates a moderate risk of bias, and 0-7 indicates a high risk of bias. MINORS: Methodological Index for Non-Randomized Studies

The Methodological Index for Non-randomized Studies (MINORS) assessment tool was used in non-randomized comparative studies. A high score indicates a low risk of bias. Two studies achieved a score of 21, two others scored 18, and one paper scored 20 (Figure 4). The MINORS has also been used in non-randomized and non-comparative studies. A low score of bias was observed, with five studies scoring 14, two scoring 13, and three scoring 12 (Figure 5).

Discussion

Cochlear implantation poses various concerns due to intraoperative and postoperative complications, ranging from minor to major complications. Major complications were defined as those requiring hospital admission and/or surgical revision, whereas minor complications can often be managed conservatively or with minor procedures [22]. This systematic review aimed to comprehensively assess complications associated with CIs in Saudi Arabia. Most of the included studies were retrospective and encompassed a total of 2314 implants. CI is indicated for patients with hearing loss. The predominant cause of hearing loss in the included studies was attributed to an unknown etiology, followed by congenital causes, and cochleovestibular malformation. This contrasts with global trends in which age-related SNHL, such as presbycusis, is the most prevalent type in adults [23]. One reason for this observation is that we did not analyze each subgroup separately, thus generalization of these data contributed to the overall trend in our sample.

CI is generally considered to be a safe surgical procedure. As with any other surgery, it presents with intraoperative complications such as CSF leakage, accidental facial nerve exposure, accidental dura exposure, bleeding, and surgical difficulties, as reported in the included studies. These complications emphasize the potential challenges encountered by surgeons during the procedure. CSF leakage is a significant concern because of its association with postoperative complications, such as meningitis. Accidental facial nerve exposure and dural exposure can lead to facial nerve paralysis and CSF leakage. This necessitates prompt intervention to minimize adverse outcomes. Additionally, intraoperative bleeding poses the risk of hematoma formation and compromises the surgical site incision, potentially prolonging the procedure and increasing the likelihood of postoperative complications.

The global complication rate was 19.9%, comprising 5% major complications and 14.9% minor complications. Minor complications were predominantly infectious in children (e.g., acute otitis media) and cochleovestibular complications in adults (e.g., tinnitus and vertigo). Major complications are primarily associated with reimplantation following revision surgery or device failure [24]. Our study supports this trend, with vestibular symptoms observed in 0.86% of patients, infections in 0.73%, and device malfunctioning or migration in 0.52%. These findings emphasize the importance of ongoing surveillance and postoperative management strategies to address both minor and major complications associated with CI to ensure optimal patient outcomes and long-term success of the procedure.

There are several techniques and interventions to manage intraoperative and postoperative CI complications, which depend mainly on the severity and type of the complications. There are several management options for intraoperative complications, including cochleostomy with complete packing of the middle ear space or covering the cochleostomy incision site with the temporalis fascia to control the CSF gusher. Lumbar drainage and fibrin glue administration are rarely used to manage CSF leakages. Intraoperative or immediate postoperative management is recommended. Some cases can be managed in an outpatient setting by using conservative measures [24-25]. To manage the surgical difficulties of CIs, preoperative imaging is necessary to plan the surgical approach, and surgery should be performed by an experienced surgeon who can modify the surgical technique according to the operative findings [26].

Accidental dural exposure is another intraoperative complication of CI. Ensuring that patients who undergo CI receive vaccination against Streptococcus pneumoniae since it is the most common organism causing meningitis in CI patients, which is due to accidental dura exposure. Maintaining a sterile surgical field and instruments, having an experienced surgeon perform the operation, and attending to the wound postoperatively ensure proper management of this complication [5]. Additionally, bleeding can also occur and is managed intraoperatively by identifying bleeding areas and controlling them by inserting a bleeding drainage plug, followed by pressure dressings to safely ensure the stoppage of the bleed [27]. Facial nerve stimulation is another complication of CI that can be managed conservatively by simply reprogramming the device or requiring re-implantation in some cases [28].

For postoperative complications, vestibular symptoms often resolve within 24 hours following surgery; however, if they persist, lifestyle changes followed by corticosteroid administration are some of the possible management options. Cochlear reimplantation and rehabilitation are considered the last line of treatment for vestibular symptoms following CIs [29]. Infection is another postoperative complication of CI. It can be managed conservatively using a standard regimen of intravenous antibiotics or by reimplantation. Minimal surgical intervention, in addition to long-term medical treatment, is preferred before re-implantation [9-30]. Device malfunction is a major complication of CI and is managed by explanation and cochlear reimplantation. Device migration when not in a normal position requires revision surgery [14].

Pain is another possible complication of CI, and it usually resolves within a few days of surgery; however, if it persists, over-the-counter analgesics may be administered [9]. In addition, facial palsy and nerve injury can occur during CI. Depending on the severity of nerve injury. Severe nerve injuries can be managed by nerve exploration, decompression, and attachment of injured nerve endings. Steroid administration is recommended if the nerve is intact. The concurrent administration of antiviral medications may also be helpful [31].

Lastly, policymakers can leverage the CI complications data on this systematic review to shape effective policies. By analyzing trends and risk factors, they can implement regulations to improve patient safety, enhance surgical procedures, and ensure better post-operative care. Early screening plays a crucial role in this framework, enabling the identification of candidates suitable for implants and assessing potential risks beforehand. Moreover, investing in early screening programs not only improves patient outcomes but also reduces long-term healthcare costs associated with complications. By integrating such insights, policymakers can foster an environment conducive to the success and safety of cochlear implant procedures in Saudi Arabia.

Limitations

This systematic review is the first comprehensive investigation of the incidence of complications following CIs in Saudi Arabia, covering a total of 2314 implants. However, some limitations must be addressed in order to interpret our results carefully. As a systematic review, our findings were reliant on the accuracy and completeness of previously published reports, all of which were retrospective in design. This retrospective nature introduces inherent biases and limitations such as potential inconsistencies in data collection and reporting. Additionally, in our database search, we retrieved published literature and therefore could not correct biases that may arise during the publication process, such as publication bias favoring studies with positive results. Also, this systematic review only reports clinical epidemiological data from Saudi Arabia, so the results of this review may not be applicable to other populations. This systematic review provides valuable insights into CI complications in Saudi Arabia. Understanding these complications is essential for optimizing patient outcomes, refining surgical techniques, and guiding future research efforts aimed at improving the safety and efficacy of CI procedures in this region.

## Conclusions

This systematic review of 2314 CIs in Saudi Arabia revealed a spectrum of intraoperative and postoperative complications associated with cochlear implantation. Addressing these complications is imperative for refining surgical techniques, optimizing patient outcomes, and advancing cochlear implantation practices in the region. This research emphasizes the importance of ongoing efforts to mitigate risks and improve the safety and efficacy of cochlear implantation procedures in Saudi Arabia. Future recommendations include conducting global epidemiological studies to determine the incidence of CI complications from around the world.
